# Remote Cerebellar Hematoma Following Supratentorial Craniotomy: A Case Report

**DOI:** 10.7759/cureus.39647

**Published:** 2023-05-29

**Authors:** Fahad M Okal, Abdulaziz Hamzah, Hani Mahboob, Riyadh Alrebai

**Affiliations:** 1 Neurosurgery Section, Department of Surgery, King Abdulaziz Medical City, National Guard Health Affairs, Jeddah, SAU; 2 College of Medicine, King Saud Bin Abdulaziz University for Health Sciences, Jeddah, SAU; 3 Department of Neurosurgery, King Fahad General Hospital, Jeddah, SAU

**Keywords:** case report, neurosurgery, glioma, intracerebellar hemorrhage, intracranial hemorrhage, supratentorial craniotomy, remote cerebellar hemorrhage

## Abstract

Remote cerebellar hemorrhage (RCH) is a rare complication following supratentorial craniotomies with unclear pathophysiology, predisposing factors, and clinical outcomes. This is a case of a 46-year-old female who presented to the emergency room with a complaint of severe headache associated with nausea. MRI studies demonstrated right frontal lesions consistent with low-grade glioma. She underwent a right frontal craniotomy, and the tumor was resected successfully. She developed a severe headache on postoperative day five, and CT scans showed ipsilateral cerebellar hematoma. She was managed conservatively and made a complete recovery within five days. Although rare, RCH requires prompt recognition, neurological monitoring, and management. Medical management and observation may be considered for patients without mass effect or acute hydrocephalus.

## Introduction

It is well-documented that the occurrence of intracranial hemorrhages in the surgical area is a common risk associated with craniotomies, surgical evacuations, and resections, including those performed in the supratentorial region of the brain [1]. Conversely, the manifestation of remote cerebellar hemorrhage (RCH), which pertains to cerebellar hemorrhage occurring beyond the surgical field, is an infrequently observed complication in neurosurgery, with documented incidence of 0.08-0.6% after supratentorial craniotomies [1]. Since its initial description by Yasargil and Yonekawa in 1977, remote cerebellar hemorrhage has captivated the neurosurgical community due to the lack of clarity regarding its underlying pathophysiology, predisposing factors, therapeutic options, and clinical outcomes [1,2]. In this case report, we present a patient who developed an ipsilateral remote cerebellar hemorrhage after a right frontal low-grade glioma resection.

## Case presentation

**Figure 1:**
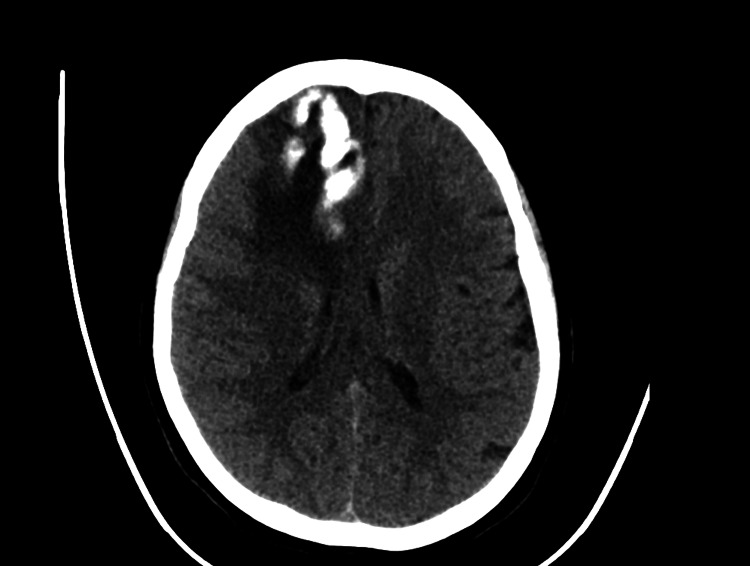
Brain CT showing hypodense right frontal subcortical lesion with calcification, vasogenic edema, and mass effect on ipsilateral frontal horn of the lateral ventricle.

**Figure 2:**
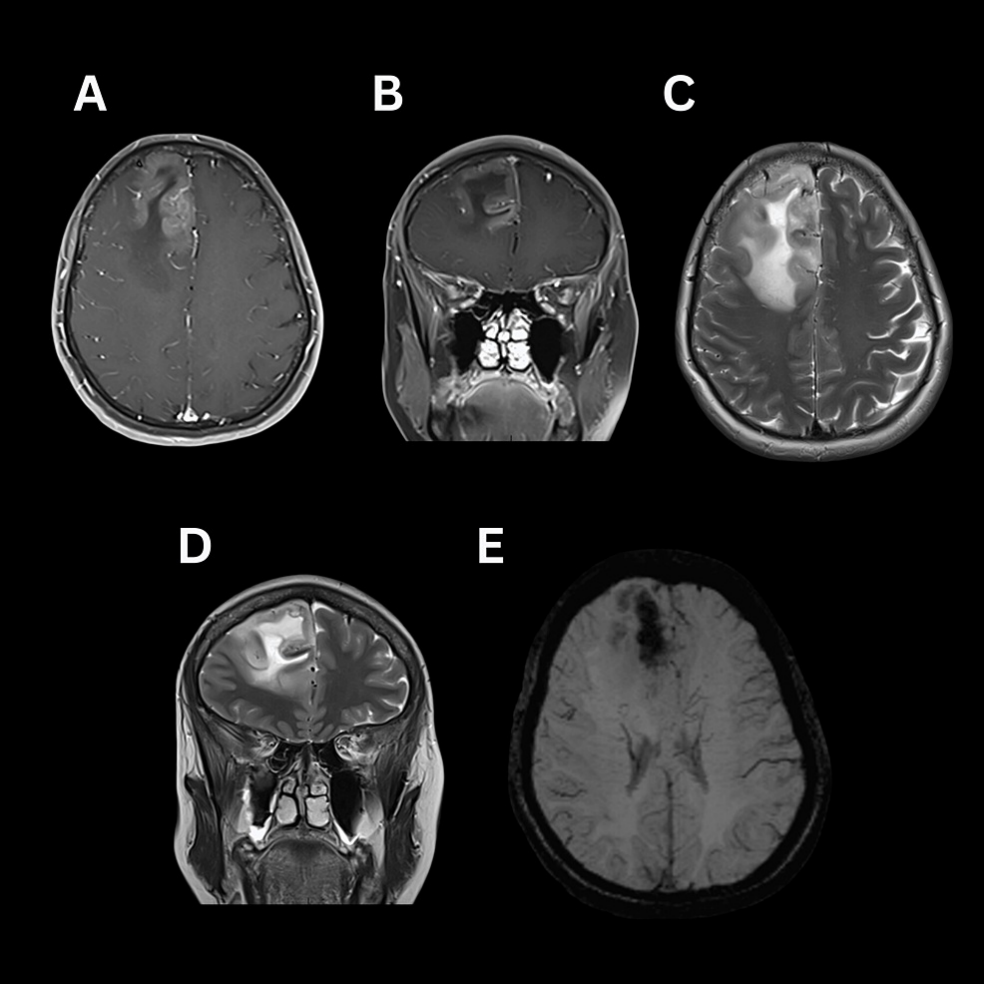
Brain MRI demonstrating hypointense lesion in T1 and hyperintense in T2, with blooming artifact in SWI (A: T1+Contrast Axial; B: T1+Contrast Coronal; C: T2 Axial, D: T2 Coronal; E: SWI). SWI: susceptibility weighted imaging

A 46-year-old female who has been a known case of controlled mass-induced seizure on levetiracetam for 20 years presented to our emergency room with a complaint of severe persistent headache-associated nausea and vomiting. The patient visited the emergency department for similar complaints three times over a period of six months. She was previously diagnosed with a brain space-occupying lesion in another institution where she was started on antiepileptic medications but refused surgical treatment. The neurological examination was unremarkable. Brain CT showed a right frontal intra-axial hypodense lesion with calcification associated with vasogenic edema (Figure 1). Brain MRI revealed that the right frontal subcortical lesion has features suggestive of oligodendroglioma. No other abnormalities have been noted (Figure 2).

A right frontal craniotomy and gross total resection of the lesion was carried out uneventfully. The ventricles were not breached during the surgery and its integrity was not compromised. Postoperatively, she was extubated in the operating theater, was vitally stable, and had no neurological deficits. Day one postoperative CT was unremarkable apart from expected postoperative changes following tumor resection, and the cerebellum was unremarkable. Histopathological examination of the surgical specimen revealed an isocitrate dehydrogenase (IDH)-mutant glioma with 1p19q codeletion on genetic studies consistent with oligodendroglioma (WHO 2).

**Figure 3:**
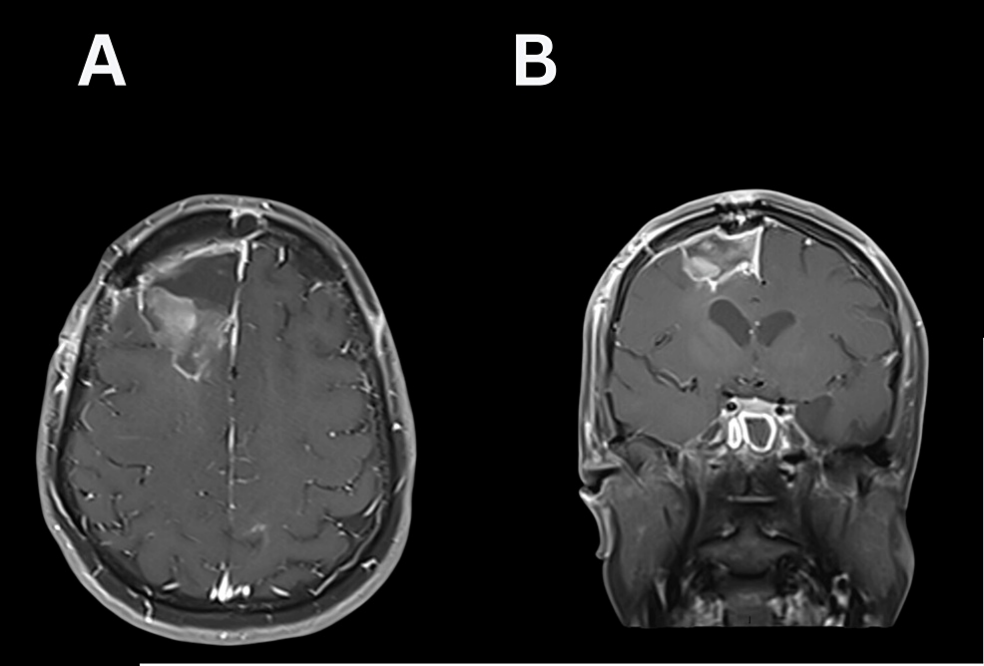
Brain MRI following gross total resection (A: T1+Contrast Axial; B: T1+Contrast Coronal).

Postoperative MRI revealed no significant decompression of the supratentorial space with no invasion of the lateral ventricles (Figure 3).

**Figure 4:**
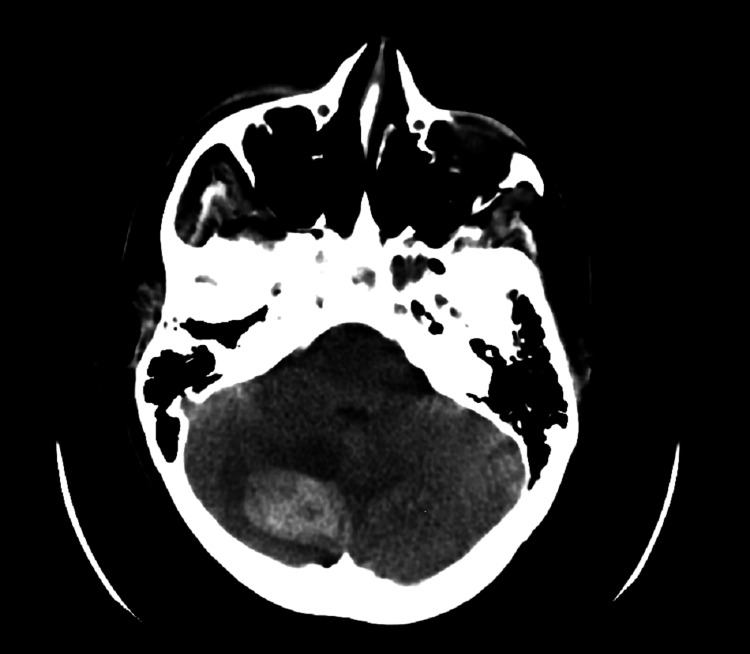
Brain CT showing a remote cerebellar hemorrhage.

Perioperative monitoring of blood pressure, coagulation, and neurological status were all normal. On postoperative day two, prophylactic subcutaneous enoxaparin was initiated. On the fifth day after surgery, the patient suddenly experienced a severe headache that was not associated with decreased level of consciousness, cerebellar signs, or seizures. During the patient's hospital stay, especially days two to five, there were no episodes of seizures or high blood pressure. Urgent brain CT scan revealed an intra-parenchymal hematoma in the right cerebellar area with surrounding cytotoxic edema with patent fourth ventricle and quadrigeminal cisterns (Figure 4).

Laboratory test results, including the coagulation profile, were normal. Conservative treatment with dexamethasone was initiated, and on the 10th postoperative day, the patient fully recovered and was discharged home.

## Discussion

RCH is a rare complication associated with supratentorial craniotomies. It is characterized by intraparenchymal cerebellar hemorrhage distant from the surgery site [1]. While RCH can occur after various types of neurosurgical procedures, it is most frequently associated with intracranial aneurysm clipping, tumor resections, and lobectomies for focal epilepsy [3]. This case report describes a patient who developed RCH following a craniotomy and resection of low-grade glioma in the right frontal lobe.

A systematic review of 209 patients conducted by Sturiale et al. (2016) found that around one-third of patients had asymptomatic RCH that was discovered incidentally in postoperative imaging. When symptomatic, patients exhibit a decreased level of consciousness and cerebellar signs, seizures, and in rare cases, coma [3]. Our patient had a sole complaint of severe headache, which is an unusual presentation of this condition. In their systematic review, 80% of patients had a clinical onset of RCH in postoperative day one. On the other hand, only 6.5% had a clinical onset after 72 hours of the surgery [3], which is the case with our patient. 

The risk factors of developing RCH are history of hypertension, coagulopathy, antiplatelet/anticoagulant use especially within a week before surgery, and perioperative complications such as seizures and hydrocephalus [3]. None of these risk factors applied to our patient as she was not a case of hypertension and was vitally stable with no episodes of seizures throughout the hospital stay. 

The symptoms of RCH may vary, and the condition is often diagnosed by neuroimaging. Patients diagnosed with RCH demonstrate distinct radiological characteristics that distinguish this form of intracerebral hemorrhage from others. These features manifest as a pattern of alternating hyperdense and hypodense curvilinear structures with irregular stripes, which resemble the distinctive coat of a zebra and have consequently been coined the "Zebra-sign" [4]. The occurrence of RCH can be either unilateral or bilateral, with vermis involvement being relatively rare [5]. The Zebra-sign is seen in 65% of RCH patients. However, in 18.5% of cases, the pattern of hemorrhage is within the cerebellar parenchyma (ICH) [3]. Our case did not exhibit classical radiological Zebra-sign but rather an intra-parenchymal cerebellar hemorrhage.

Several theories have been proposed to explain the development of this complication, including rapid loss of cerebrospinal fluid (CSF), resulting in shearing forces and tearing of the cerebellar veins [6]. Konig et al. have also suggested that a sudden decrease in intracranial pressure (ICP) leads to an increase in the transmural pressure of venules, thereby causing RCH. However, the pathomechanics of RCH remain unclear and are still subject to ongoing research. It is crucial to avoid rapid CSF drainage to prevent this complication [7].

Management of RCH depends on the clinical status and radiological findings. RCH cases are usually benign, and the majority of patients improve with observation or medical management. However, patients must be carefully monitored for any potential deterioration. Generally, older patients were correlated with poorer outcomes, while the asymptomatic presentation was associated with a favorable prognosis [3]. In some instances, RCH can be life-threatening, leading to obstructive hydrocephalus and death. Surgical interventions, such as suboccipital decompression +/- hematoma evacuation, are indicated when edema deteriorates, causing mass effect, or in cases of large hematomas or shunting in patients with obstructed fourth ventricle [8].

## Conclusions

Despite being a rare complication following supratentorial craniotomy and resection of space-occupying lesions, remote cerebellar hemorrhage necessitates prompt recognition and neurological monitoring for any signs of further deterioration. Medical management and observation should be initiated for patients without mass effect or acute hydrocephalus. Surgical decompression is to be considered when the patient's level of consciousness is decreased due to posterior fossa tightness. In cases of acute hydrocephalus, external or ventriculoperitoneal shunting may be beneficial. Timely decision-making and management are crucial to the best possible outcome. Further research is necessary to better understand the mechanisms and risk factors associated with remote hemorrhages following brain tumor resection.
